# Comparison of 2D and 3D visualization in minimally invasive and robotic surgery: a systematic review

**DOI:** 10.1007/s00464-026-12626-7

**Published:** 2026-02-04

**Authors:** Dimitrios Chatziisaak, Ismail Labgaa, Stephan Bischofberger, Dieter Hahnloser

**Affiliations:** 1 Department of Surgery, Centre Hôpitalier Universitaire Vaudois, Lausanne, Switzerland; 2 Department of Surgery, Kantonsspital St. Gallen, St. Gallen, Switzerland; 3 Department of Transplant Surgery, Guy’s and St. Thomas’ NHS Foundation Trust, London, UK

**Keywords:** Minimally invasive surgery, Three-dimensional visualization, Two-dimensional visualization, Robotic surgery, Operative time, Systematic review

## Abstract

**Background:**

Minimally invasive surgery (MIS) has transformed abdominal surgery by improving recovery times and maintaining comparable outcomes to open surgery. However, the loss of three-dimensional (3D) visualization remains a limitation. Robotic platforms have facilitated the adoption of 3D systems. Despite these developments, the advantages of 3D over two-dimensional (2D) visualization in MIS and robotic surgery remain controversial.

**Methods:**

A systematic review was conducted including studies published between January 2015 and May 2024. Studies comparing 2D and 3D visualization in MIS and robotic surgery were included. Primary outcomes assessed were operative time, intraoperative blood loss, length of stay, conversion and complication rates.

**Results:**

Regarding MIS, three studies demonstrated a statistically significant reduction in blood loss favoring 3D visualization, with median reductions from 60 mL (IQR 20–60) to 20 mL (IQR 5–40) (*p* = 0.008). Operative time was significantly reduced in 15 studies, notably in hernia repairs, bariatric, gastric, and colorectal surgeries. Two studies reported marginally significant reductions in hospital stay. Complication rates showed isolated improvements. In robotic surgery, 14 studies showed 3D visualization enhanced task performance, with tasks completed up to 88% faster among novice surgeons.

**Conclusion:**

3D visualization consistently reduces operative time in selected MIS procedures and improves robotic task performance, whereas effects on blood loss**,** complications**,** and length of stay are inconsistent. Standardized, procedure-specific trials—especially against 2D-4K—and reporting of clinical outcomes in robotic surgery are needed.

**Supplementary Information:**

The online version contains supplementary material available at https://doi.org/10.1007/s00464-026-12626-7.

Minimally invasive surgery (MIS) is a milestone in the therapeutic arsenal for nearly all abdominal surgical pathologies. It enables the provision of a safe and efficacious therapeutic surgical therapy for patients, while conferring advantages such as accelerated recovery and an expedited return to everyday activities without compromises in the surgical outcome in comparison to open surgery [1, 2]. The primary disadvantage of this technique is the loss of three-dimensional (3D) vision and the necessity for compensatory mechanisms to maintain the quality standards of the open surgery [3]. The concept of utilizing 3D visualization in laparoscopic surgery is not a novel one. However, the technological advancements that have occurred in recent years, with first and foremost the robotic surgery, have enabled its implementation in everyday clinical practice.

The advancement of robotic surgery and its inherent compatibility with 3D visualization have rendered it indispensable for most surgical procedures. The main advantage of 3D visualization is the compensatory effect for the inevitable loss of haptic feedback in the robotic surgery [4]. In recent years, there has been a growing body of research examining the potential benefits of 3D visualization in laparoscopic surgery and vice versa, the potential use of 2D visualization in robotic surgery. This research has sought to compare the use of 3D visualization with that of 2D visualization in terms of operative time, blood loss, length of hospitalization, and intra- and post-operative complications [5, 6]. The findings of retrospective comparative studies indicate improvement in some of the aforementioned factors when using the 3D visualization, though the results are not consecutive in all the conducted research [7–9]. In a recent meta-analysis comparing 2D and 3D vision in laparoscopy, Amiri et al. [10] only identified a reduction of surgery duration but no difference in other outcomes such as complications.

Unlike prior meta-analyses that primarily focused on randomized trials in laparoscopic surgery, the present review adopts a broader and complementary approach. First, it integrates contemporary randomized and non-randomized clinical studies to reflect real-world minimally invasive surgical practice across specific procedures. Second, it systematically distinguishes between clinical outcomes in MIS and performance-based outcomes in robotic surgery, incorporating robotic simulator studies. Finally, this review evaluates emerging comparisons between 3D visualization and ultra-high definition 2D systems, an increasingly relevant issue in modern surgical imaging. By doing so, this work aims to provide a clinically oriented, procedure-specific synthesis while contextualizing the role of visualization technology across both laparoscopic and robotic domains.

## Methods

The systematic review was conducted in accordance with the Preferred Reporting Items for Systematic Reviews and Meta-analyses (PRISMA) statement [11] and was registered on PROSPERO (Prospective Register of Systematic Reviews) under registration No. CRD420251041373. It was conducted in two separate steps: firstly, for MIS and secondly, for robotic surgery. The study was conducted in accordance with a pre-established protocol. Any discrepancies or disagreements between the two reviewers (D.Ch. and I.L.) at any stage of the process were resolved through discussion with one of the lead reviewers (S.B. or D.H.).

### Literature search for MIS

The MEDLINE database (via PubMed) and the Cochrane Library were searched for studies published between the dates of 1 January 2015 and 1 May 2024. The search was limited to articles published from 1 January 2015 onwards, to reflect current practice. The PICO framework was employed to define the pertinent MeSH terms [12] (Table 1). The keywords derived from the PICO table were combined with the appropriate Boolean operators to develop the search string used to identify articles in the search databases. The MeSH terms and keywords were developed for MEDLINE (via PubMed) and subsequently transformed into the Cochrane databases. To be included in the study, publications had to have undergone peer review and could be of the following types: randomized controlled trials, meta-analyses and clinical studies. It was required that the studies address the visualization modality and present data on procedural safety, technical parameters, recovery and overall surgical quality such as blood loss, operative time, length of hospitalization, conversion rates as well as postoperative complications. Only publications in the English language were included in the review. Studies published in languages other than English, in which the outcome data for different procedures were not clearly delineated, studies including general surgery procedures were excluded from the review. Studies including fewer than 15 patients were excluded to reduce the influence of underpowered analyses and extreme variability, a threshold commonly applied in systematic reviews. “General surgery procedures” were defined as abdominal and visceral operations routinely performed within general surgery practice, including hepatobiliary, gastric, colorectal, bariatric, hernia, and appendiceal procedures. All identified articles were appraised using a set of predefined appraisal criteria.

**Table 1:** PICO table to define research question and develop search string

PICO	Search strategies	Keywords (MeSH Terms + free terms)
Population	Adult patients undergoing RAS laparoscopic surgery or laparoscopic surgery	“Robotic surgical procedures[Mesh terms]” OR “laparoscopy[Mesh terms]” AND
Intervention	Surgery under 2D visualization	(“2D” OR “two dimensional” OR “two-dimensional”) AND (camera OR endoscope OR visualization)
Comparison	Surgery under 3D visualization	(“3D” OR “three dimensional” or “three-dimensional”) AND (camera OR endoscope OR visualization)
Outcome	Adverse events, complications, safety, performance, operative time, conversion	“safety” OR “adverse event*” OR “clavien-dindo” OR complications OR “blood loss” OR bleeding OR burn* OR injur* OR “performance” OR “operative time” OR “operation time” OR “procedure time” OR “console time” OR “conversion” OR comparison

### Literature search for robotic surgery

To gain further insight into potential differences performance regarding visualization modality in robotic surgery, an additional search was conducted, focusing exclusively on robotic laboratory studies. The PICO framework was employed to define the pertinent MeSH terms [12] (Table 2). The keywords derived from the PICO table were combined with the appropriate Boolean operators to develop the search string used to identify articles in the search databases.

**Table 2:** PICO table to define research question and develop search string

PICO		Keywords (MeSH Terms + free terms)
Population	Surgeons using robotic surgical system	(“davinci” OR “da vinci” OR “hugo ras” OR versius OR hinotori OR maestro OR senhance OR versius OR “robotic surgical system” OR “robotic surgery”)
Intervention	Exercise using 2D visualization	(“2D” OR “two-dimensional” OR “2D vision”) AND (camera OR endoscope OR visualization OR vision OR “imaging system”)
Comparison	Exercise using 3D visualization	(“3D” OR “three-dimensional” OR “3D vision”)) AND (camera OR endoscope OR visualization OR vision OR “imaging system”)
Outcome	Performance indicators, errors, tasks, skills, exercise	(“motor skills” OR “skills assessment” OR “surgical skills” OR “technical skills” OR “surgical task” OR task OR exercise OR “task performance and analysis” OR errors OR “suturing” OR suture OR “spatial task” OR “task complexity” OR “surgical procedure-related tasks” OR “knot tying” OR “task performance” OR “surgical performance” OR “depth perception” OR “motor task” OR “bead transfer” OR “technical skills” OR “hand–eye coordination” OR “spatial orientation” OR “instrument collision” OR “operative time” OR haptic OR “haptic feedback”)

In this instance, the inclusion criteria were peer-reviewed comparative studies utilizing robotic simulator studies. The publications had to address the visualization and tasks related to surgical procedures. All studies included were written in the English language. Systematic or narrative reviews, conference abstracts, posters and video articles were excluded from the review process. Given the paucity of available data and the limited number of subjects included in the identified publications, case series comprising a small number of subjects (i.e., greater than five) were also included in the analysis.

### Quality assessment

Methodological quality and risk of bias were assessed independently by two reviewers using validated tools appropriate to study design. Randomized controlled trials were evaluated using the Cochrane Risk of Bias 2 (RoB 2) tool, while non-randomized clinical studies were assessed using the Newcastle–Ottawa Scale. Robotic laboratory and simulator studies were evaluated qualitatively with respect to potential sources of bias, including participant selection, performance bias, and outcome assessment, given the absence of validated risk-of-bias tools for experimental task-based studies. Any disagreements were resolved by consensus.

## Results

### Visualization and MIS

A total of 1,199 records were identified across MEDLINE and the Cochrane Library. Following the removal of duplicates, irrelevant publications, publications in other languages, reviews, study protocols and conference abstracts, a total of 236 publications were subjected to screening based on their titles. A total of 125 publications were excluded from further consideration after screening the abstracts. Of the 111 remaining publications, one was excluded on the grounds that the full text could not be located. Following further screening, 48 of the remaining 110 publications were included in the review (Online Appendix 1). Of the 48 publications identified, 31 referred to general surgery procedures and were subjected to analysis. Some studies referred to multiple general surgery procedures.

**Fig. 1:**
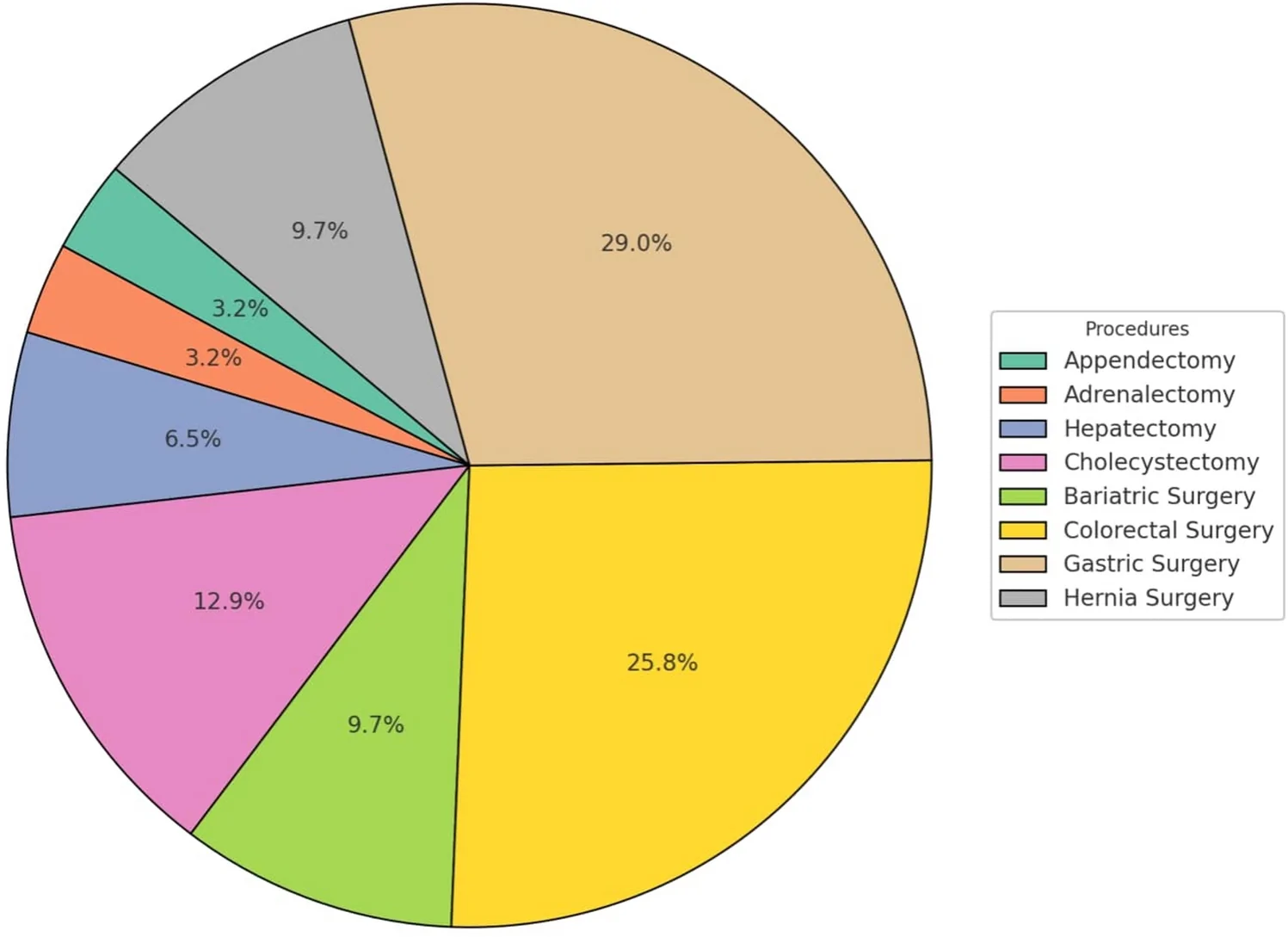
Distribution of the included studies by surgical procedure

The studies of general surgery included in this analysis were divided into subgroups according to the procedure under investigation. One of the publications refers to appendectomies [13], one to adrenalectomies [14], two to hepatectomies [15, 16], four to cholecystectomies [17–20], three to bariatric procedures [21–23], eight to colorectal surgery [24–31], nine to gastric surgery [32–40], three to hernia surgery [17, 41, 42]. Furthermore, in their publication, Parshad et al. [17] conducted a comparative analysis of the results of laparoscopic Heller myotomy for esophageal achalasia, minimally invasive cholecystectomy and minimally invasive inguinal hernia repair (TAPP). In their publication, Buia et al. [43] included a variety of surgical procedures of visceral surgery grouped into three categories according to their difficulty level (Fig. 1.)

A total of 4,824 patients’ data were subjected to analysis in the included studies. Of the total number of patients, 2,398 patients underwent surgical procedures utilizing 3D visualization. The rest of them (*n* = 2426) underwent visceral surgical procedures utilizing 2D visualization. The mean age of the 3D group was 55.91 years whereas of the 2D group 56.15 years (Online Appendix 3).

**Fig. 2:**
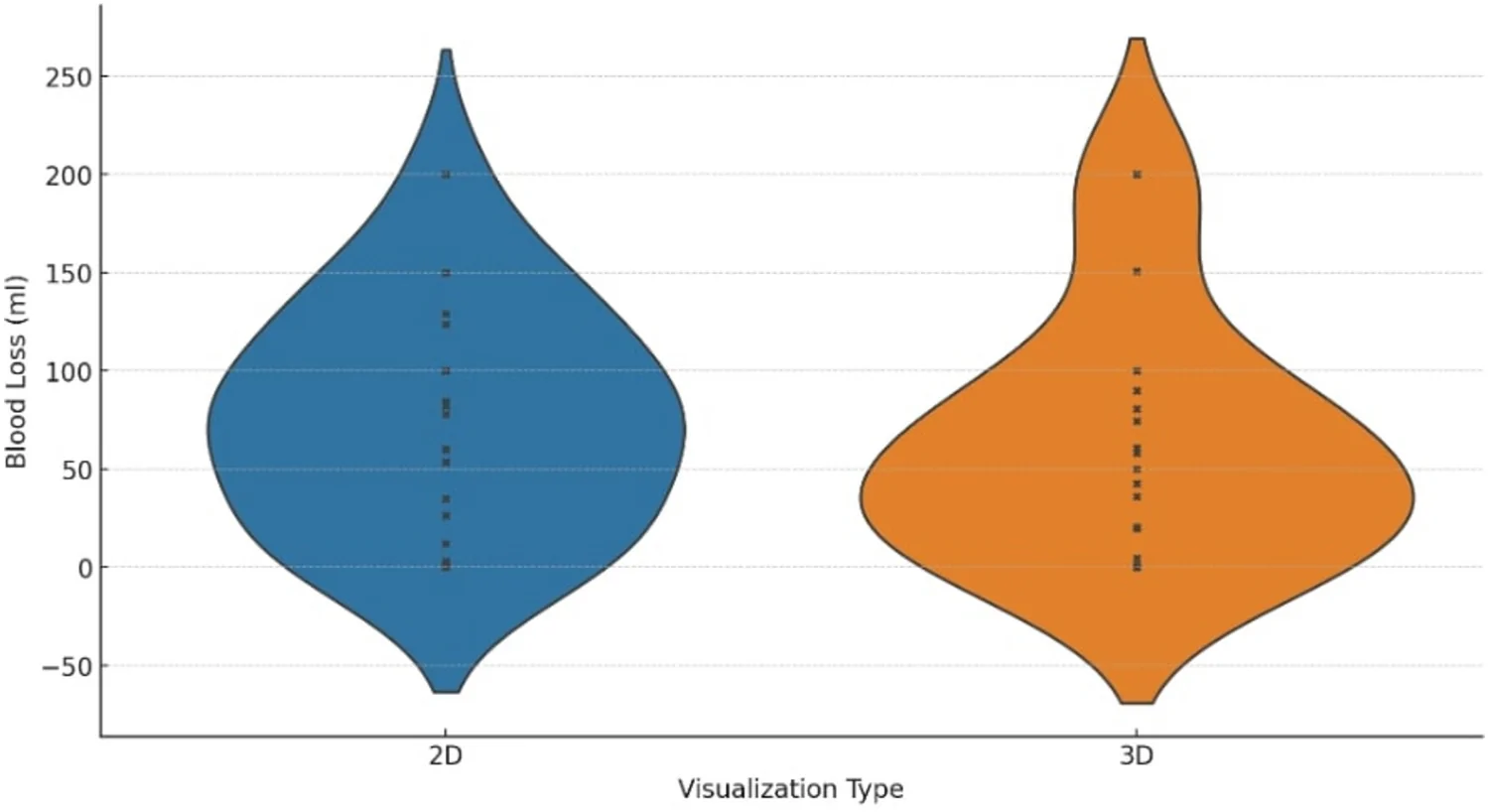
Violin plot on blood loss in 2D and 3D visualization

In total 19 of the 31 studies reported data regarding intraoperative blood loss. Most of the included data indicates only tendency for reduced blood loss when 3D visualization is employed though without reaching statistical significance (*n* = 16). Only three of the included study groups, Rodriguez-Hermosa et al. (20 mL [IQR 5–40] vs. 60 mL [IQR 20–60], 95% CI, *p* = 0.008) [14], Lin et al. (100.1 ± 48.75 mL vs 74.29 ± 47.33 mL, 95% CI, *p* < 0.001) [32], and Yoon et al. (50 mL [30–100 mL] vs 100 mL [50–100 mL], 95% CI, *p* < 0.001) [30], demonstrated a statistically significant reduction in intraoperative blood loss. It should be mentioned that the aforementioned studies reported in either of the visualization modes a maximum blood loss of 100 mL (Fig. 2). 12 studies did not report any findings concerning intraoperative bleeding or did not report the quantity of blood loss.

**Fig. 3:**
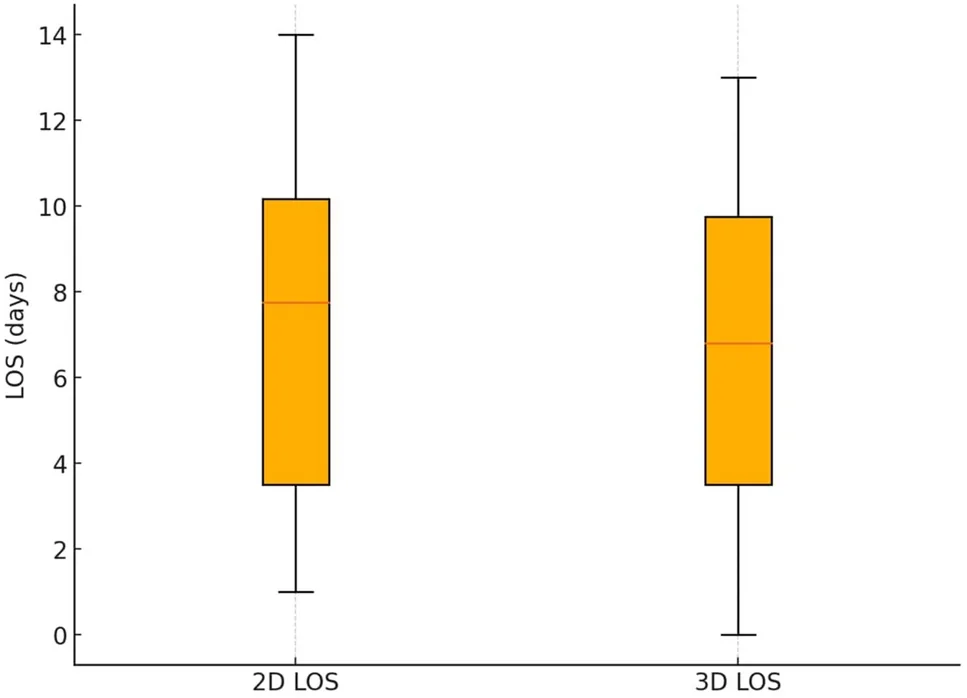
Mean length of stay (LOS) in 2D and 3D visualization

Regarding the duration of hospitalization, a statistically significant reduction in the length of stay was observed only in two study groups (2 days [range, 1–6] vs. 3 days [range, 1–11], 95% CI, *p* = 0.002) [14], (2.59 days vs 2.15, 95% CI, *p* = 0.0041) [21]. Across the studies there was a similar length of hospitalization in both modalities (Fig. 3).

Regarding the incidence of complications occurring intra- and postoperatively, Au et al. [16] observed a higher prevalence of postoperative complications in the 2D visualization cohort following laparoscopic hepatectomies for hepatocellular carcinoma (11.1 vs. 1.4%, 95% CI, *p* = 0.03). Moreover, in the study conducted by Tauriainen et al. [18], five patients (4.5%) who underwent a 2D laparoscopic cholecystectomy experienced a biliary tract injury, whereas none of the patients in the 3D visualization group sustained any intraoperative injuries. One of the included studies found a significant difference in the conversion rates between the two visualization groups (1.1% vs. 10.9%, 95% CI, *p* = 0.010) [14].

**Fig. 4:**
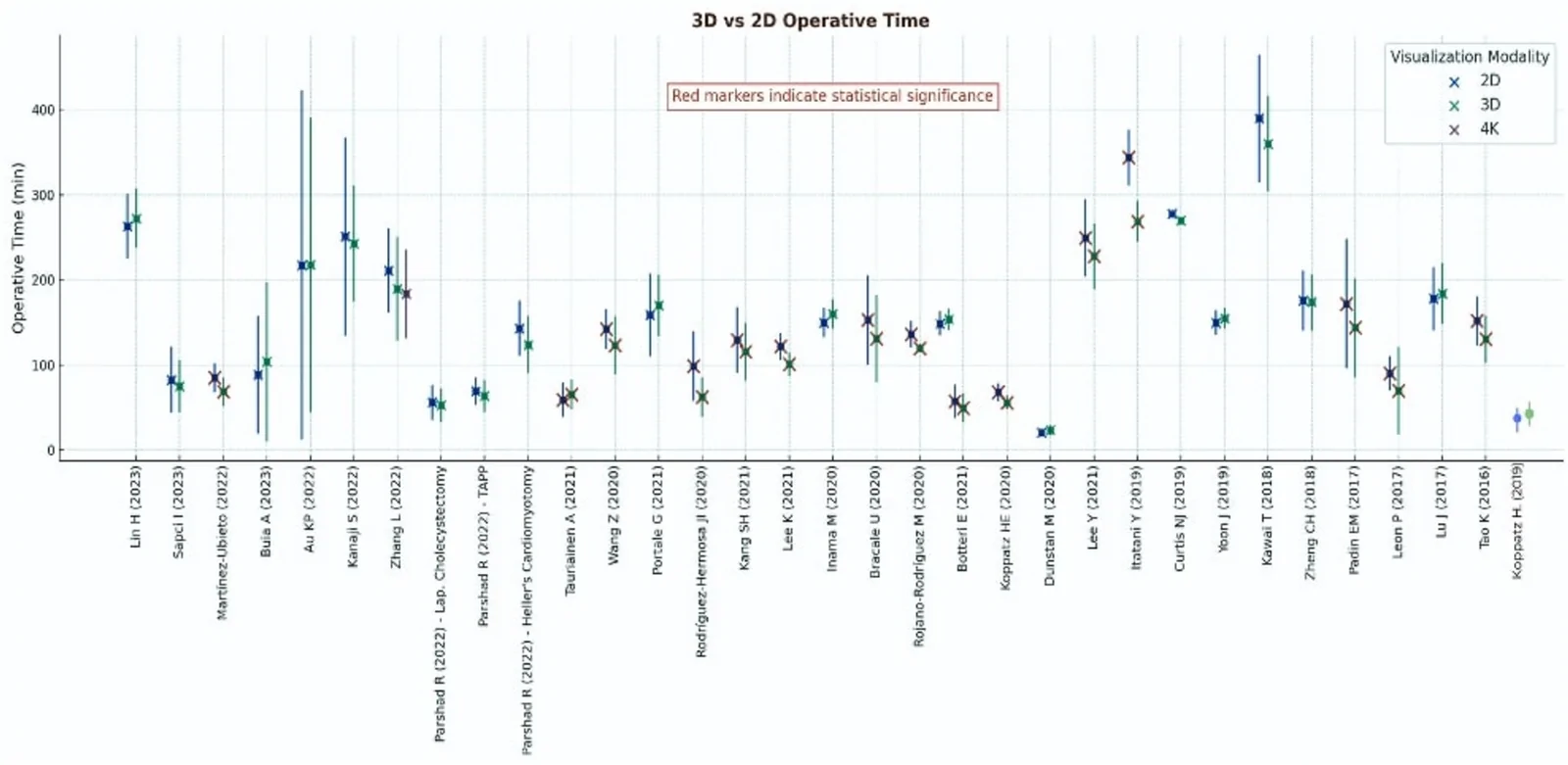
Scatter plot of operative time in the different visualization modalities

Of the studies investigating the implementation of 3D visualization in the laparoscopic hernia surgery, two of them could mark a clear significant reduction in terms of operative time (56.0 min vs. 68.0 min, 95% CI, *p* < 0.001) [41], (69.9 vs 90.1 min, 95% CI, *p* = 0.006) [42]. Although both studies concern the analysis of hernia repair, only one refers to inguinal hernia repair [41], whereas the other refers to hiatal hernia repair [42]. It is therefore evident that the distinction between the two surgical procedures precludes the possibility of drawing definitive conclusions regarding operative time. In terms of the overall surgical time, one study demonstrated a reduction in laparoscopic adrenalectomy using 3D visualization, three studies in bariatric surgery, one study examining the operating time in laparoscopic cholecystectomy and four studies in colorectal surgery, as well as four studies analyzing laparoscopic gastrectomies [14, 18, 21–23, 25, 28, 31, 35–38, 40] (Fig. 4).

A comparative analysis of 3D visualized surgical procedures with ultra-high-definition two-dimensional systems revealed no notable discrepancies between the two technologies [17, 19, 33, 34].

In summary, the evidence in the literature suggests a tendency to intraoperative reduced blood loss and reduced operating time using 3D visualization, though not always reaching statistical significance.

### Visualization and robotic surgery

A total of 371 publications were subjected to review, comprising 340 items sourced from PubMed and 31 items from the Cochrane Online Library. Six duplicates were removed. Following the title revision, 222 articles were identified as not aligning with the main subject area and were excluded. Furthermore, 17 publications were not in English and thus were excluded. 18 reviews, 6 study protocols and 10 conference abstracts were excluded. Of the remaining publications, only 14 were included in the final analysis [44–57] (Online Appendix 2).

Of the 14 publications included in the review, 12 specified that the robotic system used was the Da Vinci Robotic Surgical Systems© (Intuitive, Sunnyvale, California, U.S.), whereas two of them did not specify the robotic system used. All the included publications were concerned with the development of surgical skills, including techniques such as threading, knot tying, suturing and object placement.

Three of the included studies employed a control group to assess the performance of surgeons in threading [44–46]. The performance of the surgeons was evaluated based on the time required to complete the assigned task. The results of all the studies demonstrated that the utilization of 3D visualization can markedly reduce the surgical time required by both novice and experienced surgeons. In their study, Zwart et al. investigated the impact of 3D visualization on the performance of pancreatojejunostomy (PJ) and hepaticojejunostomy (HJ) using a bio tissue model. They compared the performance of surgeons using the Objective Structured Assessment of Technical Skills (OSATS) [58]. They observed a statistically significant reduction of 11.2 min (95% CI 6.1–15.5, *p* < 0.001) in operating time (37.2 min [IQR 30.5–43.5] vs 49.2 min [IQR 44.3–58.0]) for the PJ, and 7.2 min reduction (95% CI 4.4–13.2, *p* < 0.001) for the HJ. Moreover, the observed an improvement in the controlled skills of OSATS of 6.1 points, ± 5.5, *p* = 0.003 for both PJ and HJ [47].

Regarding suturing, the group of Tanaka et al. in their publication showed a time reduction of 9.9% using 3D visualization (161.5 s. ± 61.4 vs 145.5 s. ± 69.5, 95% CI, *p* = 0.348) [45]. In terms of object placement, threading or transfer nine studies were found and included [44–46, 48–51, 54, 55]. Six of them could prove a statistically significant time reduction in novice and experienced surgeons. Two of them could show a statistically significant difference in novice group [49, 54]. The group of Falk et al. could not find any statistically significant difference in time of completion, when 2D-high-resolution technology was compared to 3D visualization in suturing (77 s. ± 24 vs. 68 s ± 15, 95% CI, *p* = 0.55) and knot-tying (58 s ± 20 vs. 41 s. ± 22, 95% CI, *p* = 0.41) [50].

The results of three of the included studies demonstrated that task completion occurred in a shorter time in the robotic setting, irrespective of the visualization technique, in comparison to the laparoscopic setting [51, 52, 57].

Most RCTs were judged as low to some concerns of bias, primarily due to performance blinding limitations. Observational studies generally achieved moderate NOS quality, with the main limitation being selection bias related to retrospective design.

## Discussion

In this systematic review, we compared the data of studies including more than 4500 patients to evaluate the efficiency and safety of 2D and 3D visualization in MIS, taking under consideration the data of the latest meta-analysis [10]. Additionally, we performed an inaugural data analysis from the 14 published studies that have hitherto compared the visualization modalities in robotic surgery.

The present review should be interpreted as complementary to, rather than duplicative of, existing meta-analyses such as that by Amiri et al. [10]. By separating minimally invasive clinical outcomes from robotic task-based studies, the current review expands the analytical framework to include visualization-dependent skill acquisition, learning curves, and technical performance—domains that are increasingly relevant in modern surgical training and practice.

As indicated by the most recent published meta-analysis of Amiri et al. [10], the sole statistically significant benefit associated with the utilization of 3D technology in MIS appears to be a reduction in surgical time (− 8.0%; SMD, − 0.22; 95% CI, − 0.37 to − 0.06; *p* = 0.007; *n* = 3003; 24 studies; *I* ^2^ = 75%). In our review we confirm that the use of 3D visualization in MIS has been demonstrated to reduce operating time first and foremost in hernia procedures, gastrectomies, bariatrics and colorectal procedures.

Our systematic literature review revealed three studies that reported a statistically significant reduction in intraoperative blood loss when 3D visualization was employed in MIS. In contrast, most of the literature merely indicated a tendency for reduced bleeding, though this was not statistically significant. This discrepancy is evident also in the reported results of the two large meta-analyses conducted by Amiri et al. [10] and Liang et al. [53]. The latter group identified a statistically significant reduction of the intraoperative blood loss (SMD − 0.26; 95% CI − 0.49 to − 0.03; *p* = 0.028, *I*^2^ = 87.2%, *p* =  < 0.001).

Regarding the use of 2D visualization in the robotic surgery, the results of the included studies demonstrated a statistically significant improvement in task performance when 3D visualization was employed when compared to 2D.

The degree of improvement in task performance was less pronounced in tasks that were perceived as easier to complete. The performance of suturing on a simulated skin incision was observed to be 31% faster under 3D visualization, while the more complex S-hook passage was found to be 88% faster under the same conditions [51]. All participants perceived the tasks as more challenging in 2D visualization and reported reduced stress levels with the enhanced 3D visualization. Both dexterity and accuracy were enhanced under 3D visualization [48].

The performance of novice users with no or limited experience in surgical procedures and expert users with substantial experience in laparoscopic or robotic surgical procedures was also compared under 2D and 3D visualization. The novice groups demonstrated inferior performance under 2D visualization compared to the expert users [46, 54, 55]. The learning curve was found to be shorter under 3D visualization. This indicates that surgeons with greater experience in surgical procedures conducted in 2D environments, such as in the 2D MIS, may find the loss of depth perception less challenging to adapt to. It is noteworthy, however, that the study by Blavier et al. [54] indicated that the performance of the expert surgeons was found non inferior under 2D visualization, which could potentially diminish the influence of the visualization mode on performance, given the expertise of the surgeon in each given surgical task. Because of that, we believe that greater the proficiency in performing a surgical task in robotic surgery, the less important the visualization may be. It is noteworthy that most of the included studies had a small sample size, which limits the statistical impact of the results. The study by Tanaka et al. did not demonstrate a statistically significant difference between 2 and 3D for the novice group, while the analysis included only three expert users and three novices [45]. Studies with a larger sample size demonstrated significant differences between the novice and expert groups [44, 51]. These findings should be interpreted in the context of procedure-specific technical complexity, surgeon experience, visualization technology, and study design, all of which are discussed above and appear to modify the observed effects of 3D visualization.

Although the physiological rationale for 3D visualization is intuitive, the clinical impact observed in contemporary studies appears modest. Across most included studies, improvements were primarily limited to small reductions in operative time, while differences in blood loss, length of stay, conversion rates, and postoperative complications were inconsistent or absent. These findings suggest that, for many procedures, 3D visualization may offer incremental rather than transformative clinical benefit.

Importantly, the rapid adoption of ultra-high-definition 2D systems, including 2D-4 K technology, has narrowed the performance gap traditionally observed between 2 and 3D visualization. Several studies included in this review reported comparable outcomes between 3D systems and modern 2D-4 K platforms, underscoring that image resolution and visual clarity may partially compensate for the lack of stereoscopic depth perception in routine clinical practice.

Although robotic surgery under 3D visualization is associated with better overall performance, users also demonstrated superior outcomes in robotic surgery under 2D visualization when compared to minimally invasive laparoscopic surgery, though these results were not consistently statistically significant. Further prospective studies are required to address this question in the context of robotic surgery.

Several limitations should be acknowledged. A substantial proportion of the included studies were non-randomized and therefore subject to inherent risks of selection and performance bias, particularly in procedure-specific retrospective analyses. An important consideration in interpreting these findings is the substantial heterogeneity in technical complexity and depth-perception demands across different surgical procedures. Operations such as hepatectomy, gastrectomy, and colorectal resections require precise spatial orientation, complex dissection planes, and advanced intracorporeal maneuvers, which may explain the more consistent operative time reductions observed with 3D visualization in these settings. In contrast, procedures with more limited spatial complexity, such as appendectomy or standard cholecystectomy, demonstrated less pronounced or inconsistent benefits. For this reason, results were intentionally synthesized in a procedure-specific manner rather than pooled across fundamentally different operations.

## Conclusion

This review demonstrates that 3D visualization in MIS offers distinctive advantages, particularly in reducing operative time and enhancing spatial awareness, which is especially beneficial in robotic and laparoscopic procedures. However, its impact on other outcomes, such as blood loss and complication rates, is less consistent. Further research is necessary to better understand the long-term clinical outcomes of using 3D visualization in diverse surgical settings.

## Supplementary Information

Below is the link to the electronic supplementary material.

Supplementary file1 (DOCX 244 KB)

Supplementary file2 (DOCX 210 KB)

Supplementary file3 (DOCX 73 KB)
