# Supplementary material for: Comparison of 2D and 3D visualization in minimally invasive and robotic surgery: a systematic review
Source: Surg Endosc. 2026 Feb 4;40(3):1828–36. doi: 10.1007/s00464-026-12626-7 (PMC13668924; doi:10.1007/s00464-026-12626-7)
Supplement: Supplementary file 2 [file 464_2026_12626_MOESM2_ESM.docx]

Appendix 2. PRISMA flow chart with details of the literature search and appraisal process in robotic surgery.
