# Supplementary material for: Comparison of 2D and 3D visualization in minimally invasive and robotic surgery: a systematic review
Source: Surg Endosc. 2026 Feb 4;40(3):1828–36. doi: 10.1007/s00464-026-12626-7 (PMC13668924; doi:10.1007/s00464-026-12626-7)
Supplement: Supplementary file 3 [file 464_2026_12626_MOESM3_ESM.docx]

Appendix 3. Summary of included articles including demographic data, study specifications and outcome data for surgeries with 2D, 3D and 2D4K visualization techniques

| **Title, Author (year)** | **Sample size** | **Surgery** | **Operative time (SD/IQR)** | **Blood loss ml (SD)** | **IOC n (%)** | **POC n (%)**  **(30 days)** | **LOS (days)** |
| --- | --- | --- | --- | --- | --- | --- | --- |
| **Lin H et al.**  **(2023)(32)** | 2D, n=119 | Gastrectomy | (total) 263.14  (± 38.2)  (distal)239.48  (±37.11) | **100.17**  **(±48.747)** | N/A | 30(25.2) CD III-V 2(1.7) | 13(11-15) |
|  | 3D, n=91 |  | 272.33 (total)  (± 34.77) 251.85 (distal)  (± 32.94) | **74.29**  **(±47.33)** | N/A | 22(24.2) | 10(9-11) |
| **Sapci I et al. (2023)(24)** | 2D, n=26 | Colorectal | 82.7(±38.6) | 53.5(±55.3) | N/A | *8 (30.8)* | 3 |
|  | 3D, n=27 |  | 75.3(±30.8) | 36.2(±20.9) | N/A | *8 (29.6)* | 3 |
| **Martínez-Ubieto**  **et al.**  **(2022)(21)** | 2D, n=37 | Bariatrics | **85(±16.8)** | N/A | N/A | 8(21.6) CD III-V 4(10.8) | **2.59**  **(±0.64)** |
|  | 3D,n=41 |  | **69(±16.9)** | N/A | N/A | 6 (14.6) CD III-V 3(7.3) | **2.15**  **(±0.65)** |
| **Buia A et al. (2023)(43)** | 2D,n=213 | Varia | 88.60(±69.32) | N/A | N/A | N/A | N/A |
|  | 3D,n=213 |  | 103.98(±93.26) | N/A | N/A | N/A | N/A |
| **Au KP et al. (2022)(16)** | 2D,n=72 | Hepatectomy | 217.5 (45-756) | 200 | N/A | **8 (11.1)** | 5(2-35) |
|  | 3D,n=72 |  | 218 (45-644) | 200 | N/A | **1 (1.4)** | 5(2-14) |
| **Kanaji S et al.**  **(2022)(33)** | 2D,n=21 | Gastrectomy | 251(196-599) | 0(0-150) | 0 | *6 (29)* | 13(8-46) |
|  | 3D,n=21 |  | 243(167-403) | 0(0-50) | 0 | *3 (11)* | 13(10-23) |
| **Zhang L et al.**  **(2022)(34)** | 2D,n=30 | Gastrectomy | 211.00(± 49.33) | 129(±103.57) | 0 | 8 (26.7) | 10.53 (±3.83) |
|  | 3D,n=32 |  | 189.69(± 60.87) | 150(±105.46) | 0 | 6 (18.75) | 10.75 (±4.71) |
|  | 4K, n=25 |  | 183.60(± 52.5) | 123(±119.51) | 0 | 4 (16.00) | 11.24 (±6.50) |
| **Parshad R et al.**  **(2022)(17)** | 4K, n=30 | CHE | 56.2(± 20.6) | N/A | 0 | N/A | N/A |
|  | 3D, n=30 |  | 52.7(±19.5) | N/A | 0 | N/A | N/A |
|  | 2D 4K,  n=24 | TAPP | 69.6(±16.4) | N/A | 0 | N/A | N/A |
|  | 3D,n=25 |  | 63.8(±18.5) | N/A | 0 | N/A | N/A |
|  | 2D 4K,  n=15 | Heller Myotomy | 143.3(±32.5) | N/A | 0 | N/A | N/A |
|  | 3D,n=15 |  | 124(±33.5) | N/A | 0 | N/A | N/A |
| **Tauriainen A et al. (2021)(18)** | 2D,n=111 | CHE | **59.3 (±20.1)** | N/A | **5(4.5)** | N/A | 2.3(±2.7) |
|  | 3D,n=130 |  | **65.4 (±17.8)** | N/A | **0** | N/A | 2.8(± 6.5) |
| **Wang Z**  **et al. (2020)(25)** | 2D,n=60 | Colorectal | **142.2 (±23.5)** | 42.5(±10.4) | N/A | 5 (8.3) | 7.8(±3.2) |
|  | 3D,n=60 |  | **123.2 (±34.2)** | 38.8(±13.4) | N/A | 4 (6.7) | 7.6(±2.5) |
| **Portale G et al.**  **(2021)(26)** | 2D,n=55 | Colorectal | 159 (±48.8) | N/A | 0 | 15 (27.3) | 9.1(±3.3) |
|  | 3D,n=59 |  | 170 (±36) | N/A | N/A | 12 (20.3) | 8.4(±2.6) |
| **Rodríguez-Hermosa JI, et al.(2020)(14)** | 2D,n=55 | Adrenalectomy | **98.6 (±40.8)** | **60(20-60)** | 0 | 2 (3.6) | **3(1-11)** |
|  | 3D,n=95 |  | **62.6 (±23.2)** | **20(5-40)** | N/A | 2 (2.1) | **2(1-6)** |
| **Kang SH et. al. (2021)(35)** | 2D,n=73 | Gastrectomy | **129.4 (±38.5)** | 35.1(±56) | N/A | 4 (4.4)  2 (2.2) CD III-V | 6.2(4-24) |
|  | 3D,n=73 |  | **115.6 (±34)** | 20.7(±30) | N/A | 2 (2.2)  CD III-V 1(1.1) | 5.4(3-12) |
| **Lee K et al.**  **(2021)(36)** | 2D,n=41 | Gastrectomy | **122**  **(106.5-161)** | 26.4(±3.5) | N/A | 10 (24.4) | N/A |
|  | 3D,n=38 |  | **101**  **(77.75-125.5)** | 19.8(±2.9) | N/A | 3 (7.9) | N/A |
| **Inama M et al.**  **(2020)(27)** | 2D,n=82 | Colorectal | 150(120-180) | N/A | N/A | 23 (28.05)  CD III-V 7(8.5) | N/A |
|  | 3D,n=231 |  | 160(130-190) | N/A | N/A | 52 (22.51)  CD III-V 21(9) | N/A |
| **Bracale U et al.**  **(2020)(28)** | 2D,n=53 | Colorectal | **153.2 (±52.4)** | N/A | 0 | *15 (28.3)  CD III-V 4(7.5)* | 6 ± 1 |
|  | 3D,n=53 |  | **131(±51)** | N/A | 0 | *12 (22.6)  CD III-V 4(7.5)* | 6 ± 2 |
| **Rojano-Rodríguez**  **et al.(2020)(23)** | 2D,n=18 | Bariatrics | **136.5(117-171)** | N/A | N/A | N/A | N/A |
|  | 3D,n=20 |  | **120(112.5-129)** | N/A | N/A | N/A | N/A |
| **Botteri E**  **et al. (2021)(13)** | 2D,n=71 | Appendectomy | 57.5 (±19.9) | N/A | 0 | 0 | 1(±11.1) |
|  | 3D,n=64 |  | 49.6 (±16.9) | N/A | 0 | 0 | 0 |
| **Koppatz HE**  **et al.(2020)(41)** | 2D,n=137 | TAPP | **68.0**  **(50.3–85.8)** | N/A | *7 (5.1)* | 24 (19.2) CD III-V 1(0.7) | N/A |
|  | 3D,n=135 |  | **56.0**  **(43.0–71.0)** | N/A | *12(8.9)* | 32 (25.2) | N/A |
| **Dunstan M et al.**  **(2020)(19)** | 2D4K n=55 | CHE | 20.90  (17.67–33.03) | N/A | N/A | 5 | N/A |
|  | 3D,n=54 |  | 23.41  (17.00–37.98) | N/A | N/A | 8 | N/A |
| **Lee Yet al.**  **(2021)(36)** | 2D,n=34 | Gastrectomy | **249.6 (45.3)** | N/A | N/A | 0 | N/A |
|  | 3D,n=34 |  | **227.8 (39.0)** | N/A | N/A | 0 | N/A |
| **Itatani Yet al. (2019)(38)** | 2D,n=40 | Gastrectomy | **344**  **(288–402)** | 3(0–40) | N/A | 6 (15) | 14(12–19) |
|  | 3D,n=29 |  | **269**  **(243–326)** | 0(0–0) | N/A | 2 (6.9) | 13(11–14) |
| **Koppatz H et al. (2019)(20)** | 2D,n=104 | CHE | 48.0  (38.5–59.8) | 11.9(±60.9) | N/A | 22 (22.7) | N/A |
|  | 3D,n=105 |  | 49.0  (39.0–65.0) | 4.8(±13.3) | N/A | 21 (22.3) | N/A |
| **Curtis NJ et al. (2019)(29)** | 2D,n=37 | Colorectal | 278 | 60 | N/A | (59.50) | N/A |
|  | 3D,n=40 |  | 270 | 90 | N/A | 62.70% | N/A |
| **Yoon J et al. (2019)(30)** | 2D,n=167 | Colorectal | 150  (125–175) | **100**  **(50–100)** | 13 (7.8) | N/A | 6(6-8) |
|  | 3D,n=111 |  | 155  (135–177.5) | **50**  **(30–100)** | 9 (8.1) | N/A | 6(6-7) |
| **Kawai T et al. (2018)(15)** | 2D,n=45 | Hepatectomy | 390 (± 75) | 150  (100-700) | N/A | 17 (38)  CD III-V 6(13) | 8(5-51) |
|  | 3D,n=30 |  | 360 (± 56) | 100  (100-600) | N/A | 12 (40) CD III-V 4(13) | 9(5-26) |
| **Zheng CH et al. (2018)(39)** | 2D,n=208 | Gastrectomy | 176 (± 35) | 82(±119) | N/A | 29 (13.9) CD III-V 5(2.4) | N/A |
|  | 3D,n=211 |  | 174 (± 33) | 61(±83) | N/A | 36 (17.1) CD III-V 5(2.4) | N/A |
| **Padin EM et al. (2017)(22)** | 2D,n=208 | Bariatrics | **172.11**  **(± 76.11)** | N/A | N/A | (4.30) | 7.7(±13.2) |
|  | 3D,n=104 |  | **144.07**  **(± 58.07)** | N/A | N/A | (1) | 5.12(±9.6) |
| **Leon P et al. (2017)(42)** | 2D,n=17 | Hiatal Hernia | **90.1 (± 19.9)** | N/A | 2 (20) | N/A | N/A |
|  | 3D,n=19 |  | **69.9 (± 51.5)** | N/A | 1 (8) | N/A | N/A |
| **Lu J et al.**  **(2017)(40)** | 2D,n=112 | Gastrectomy | 178 (± 37) | 78(±72) | N/A | 18 (16.1) | 12.63(±7.32) |
|  | 3D,n=109 |  | 184 (± 36) | 58(±75) | N/A | 20 (18.3) | 12.52(±4.83) |
| **Tao K et al.**  **(2016)(31)** | 2D,n=31 | Colorectal | **152.2 (± 28.9)** | 84.7(±22.3) | N/A | 3 (9.7) | 9 (6-21) |
|  | 3D, n=27 |  | **130.5 (± 27.6)** | 80.8(±29) | N/A | 4 (14.8) | 8 (6-14) |

Results in bold indicate statistically significant differences (p<0.05) . Sample Size is categorized by visualization 2D vs 3D. Operative Time is in minutes (min.). IOC=Intraoperative Complications. POC = Postoperative Complications. CD= Clavien-Dindo Classification. LOS= Length of Stay (Hospitalization). CHE= Cholecystectomy.
